# Inducing sad recognition bias: A novel emotional probabilistic reward task and its affective consequences

**DOI:** 10.1371/journal.pone.0291979

**Published:** 2024-11-07

**Authors:** Seongbo Lee, Sang Hee Kim

**Affiliations:** Department of Brain and Cognitive Engineering, Korea University, Seoul, Korea; University of Valencia: Universitat de Valencia, SPAIN

## Abstract

Recognition of sadness from facial expressions is associated with empathic responses. In this study, we devised an emotional probabilistic reward task (PRT) to facilitate sadness recognition and tested its effects on attentional and empathic responses to others in distress. During the emotional PRT, healthy participants were asked to discriminate between facial expressions subtly expressing sadness or anger. Reward feedback for correct sadness and anger recognition was provided, with different probabilities between the training (70% vs. 30%) and control groups (50% vs. 50%). Subsequently, participants performed a visual dot-probe task involving facial expressions of sadness, anger, fear, and happiness. They also completed an empathy rating task while viewing short video clips depicting people experiencing distressing or neutral events. The results showed that the training group developed greater recognition bias for sadness than the control group. Within the training group, sad recognition bias was positively associated with subsequent attentional orienting to sad faces and empathic concern towards distressed others. These findings suggest that the emotional PRT holds promise for modifying cognitive and emotional processes that are associated with empathy for others.

## Introduction

Recognizing emotions from facial expressions is a central skill in social cognition and critically influences our emotional states and behavior [1]. Although facial emotion recognition is believed to be universal at least for some basic emotions [2], there are substantial individual differences in emotion recognition from facial expressions. Namely, individuals may vary in their perceptual sensitivity to the emotional cues displayed on the faces and in their tendency towards specific emotions when there is lack of certainty [3–5]. These individual differences in facial emotion recognition are associated with the emotional states of the perceivers [5–7] and evident for more subtle and nuanced facial expressions than for prototypical expressions [8].

Previous research suggests that better facial emotion recognition is associated with trait empathy [9–11]. In a study involving 137 healthy individuals, individuals with high trait empathy were more accurate than those with low trait empathy in recognizing facial emotions of sadness, anger, fear, and happiness, and tended to categorize neutral expressions as sad or fearful [10]. Individuals with high trait empathy also reported strong feelings of sadness and being moved when listening to unfamiliar sad music [12]. In general, evidence supports that people with a high level of empathy recognize faster and more accurately facial expressions that signal negative emotions. Moreover, children with psychopathic tendencies, who are typically characterized by a lack of empathy, were found to have a selective impairment in the recognition of sad and fearful expressions [13]. Given the importance of decoding facial cues to understand others’ feelings and intentions, the close association between facial emotion recognition and trait empathy is comprehensible.

On the other hand, experimental manipulation to engage participants to sad faces increases empathic and prosocial responses towards others [14, 15]. In a study that instructed participants to take the perspective of a person who reacted with sadness in response to a physical illness, participants showed greater empathic concern and helping intentions toward the person than when they took the perspective of an ill person who reacted with anger and disgust [14]. In another study, participants performed a task requiring monetary allocation between themselves and another recipient. Participants sent more money to the recipient expressing facial emotions of sadness or happiness than to those expressing facial emotions of anger or disgust [15]. According to the cognitive theories of emotions [16], sad expressions signal a lack of resources to cope with goal frustration. Therefore, a proper appraisal of such signals may typically evoke empathic concern for the sad expressers, which, in turn, increase helping behavior in the perceivers [14, 17].

Provided the close association between recognition of sadness and empathic responses, we attempted to test whether experimental manipulation to facilitate sadness recognition would increase empathic responses to others in distress. To accomplish this, we first developed an association learning task designed to enhance sadness recognition from ambiguous facial expressions. This new task was adapted from the original probabilistic reward task (PRT) [18]. The PRT requires participants to discriminate between two stimuli that are ambiguous about their identity. Unbeknownst to the participants, correct identification of one stimulus is rewarded more frequently than the other. As the learning trials progress, participants typically develop systematic response preferences for a stimulus that is rewarded more frequently than the other [18, 19]. The original PRT uses a physical feature such as the length of a line (i.e., long mouth versus short mouth) as the target of discrimination. We created an emotional version of the PRT in which the emotion category is the target of discrimination. Specifically, we used ambiguous facial expressions of emotions that were minimally charged for sadness or anger as stimuli for participants to discriminate. Correct identification of ambiguous sad faces was rewarded more frequently than correct identification of ambiguous angry faces. Similar to the results from the original version of the PRT [18, 19], we expected that there would be systematic recognition preference for sadness as it was the more frequently rewarded response over the other. We contrasted sadness against anger. They both signal goal frustration, but they differ in their indication of coping abilities. Sadness signals that the expresser lacks the resources to cope [16], which may evoke empathic concern and a willingness to help in observers [15].

Experimentally induced preference for sad recognition through the emotional PRT may accompany preferential assignment of attentional resources to sad faces. From the perspective of cognitive information processing, perception and recognition of emotion is tightly associated with attention processes. Research evidence shows that biased attention to emotionally negative information (i.e., threatening faces or words) is associated with increased tendency to perceive ambiguous facial expressions to be more negative in typically developing children [20] and adults [21]. In the current study, we assessed attention allocation to facial expressions of emotions using the visual dot-probe task [22]. This task allows the assessment of early attention orienting to and disengagement from an emotional expression. We also examined empathic concern and helping intentions toward others in distress. Given the close association between sad recognition and state- and trait-related empathic responses [10, 12, 14, 15], we expected that the emotional PRT would result in increased empathic concern and helping intentions towards others.

### Overview of the present study

In the current study, healthy volunteers participated and were randomly assigned to either the training group or control group. All participants performed the emotional PRT. The task presented participants with facial expressions that depicted weak expressions of sadness or anger. The groups differed in reward contingency: the training group received reward feedback for 70% of the responses that accurately identified sad faces and 30% of responses that accurately identified angry faces. The control group received equal probabilities of reward feedback (i.e., 50% vs. 50%) for responses that accurately identified sad and angry faces. Following the emotional PRT, all participants performed the visual dot-probe task that involved presenting facial expressions of emotions, including sadness, anger, fear, and happiness. Then, participants performed an empathy rating task while viewing short video clips depicting people experiencing distressful or neutral events. Participants rated their feelings of empathic distress, empathic concern, and helping intentions for the victim depicted in each video clip. We expected that the training group would develop a systematic preference for sadness recognition relative to anger recognition as compared with the control group. The training group was also expected to show faster attention allocation towards sad faces and more empathic concern and helping intentions toward distressed victims than the control group.

## Method and materials

### Participants

A total of 74 right-handed healthy participants were recruited through online community advertising through institution and locally posted flyers throughout the community. All volunteers were college students at the time of participation. The sample size was estimated primarily to detect group differences in outcome task performance following training. Due to the novelty of our intervention manipulation, there was a lack of comparable studies to derive an effect size for estimating the sample size. Thus, we relied on findings from a prior study conducted in our lab [23]. This earlier study showed that an intervention task designed to implicitly promote positive interpretation of ambiguous facial expressions increased the likelihood of evaluating subsequently presented surprise facial expressions as positive, with an effect size of Cohen’s *d* = .71. With an effect size of 0.71, an alpha level of 0.05 and a power of 0.85 yielded a minimum sample size of 37 participants per group using G*Power software version 3.1.9.4 [24]. Participants were randomly assigned to one of the two groups: training group and control group. All participants provided written informed consent prior to their participation. The study protocol was reviewed and approved by the Korea University Institutional Review Board and performed in accordance with the ethical standards in the Declaration of Helsinki. All participants received monetary compensation for their time. Three participants were removed from the data analysis due to too many missing or outlier trials during training (> 25% of total trials, n = 2), or voluntary withdrawal from the study (n = 1). Outlier trials were defined as those having response time less than 200 ms as they were assumed not to accurately reflect signals based on the recognition decision process. This criterion is consistent with previous studies analyzing response times from the PRT [25, 26] and others involving choice-task response times [27]. Therefore, a total of 71 participants (39 females) were included in the data analyses. Data collection took place until August 2021. No personal identification information was linked to the data, and authors did not have access to any information that could identify individual participants during or after data collection.

### A novel emotional probabilistic reward task (PRT)

The intervention task was created using facial expressions of subtle emotional intensity. Specifically, two morphed facial expressions were used, each exhibiting 15% sadness or anger. They were created by morphing a neutral expression with expressions of 100% sadness and anger from the same face model selected from the standardized database ChaeLee face set [28].

**Fig 1:**
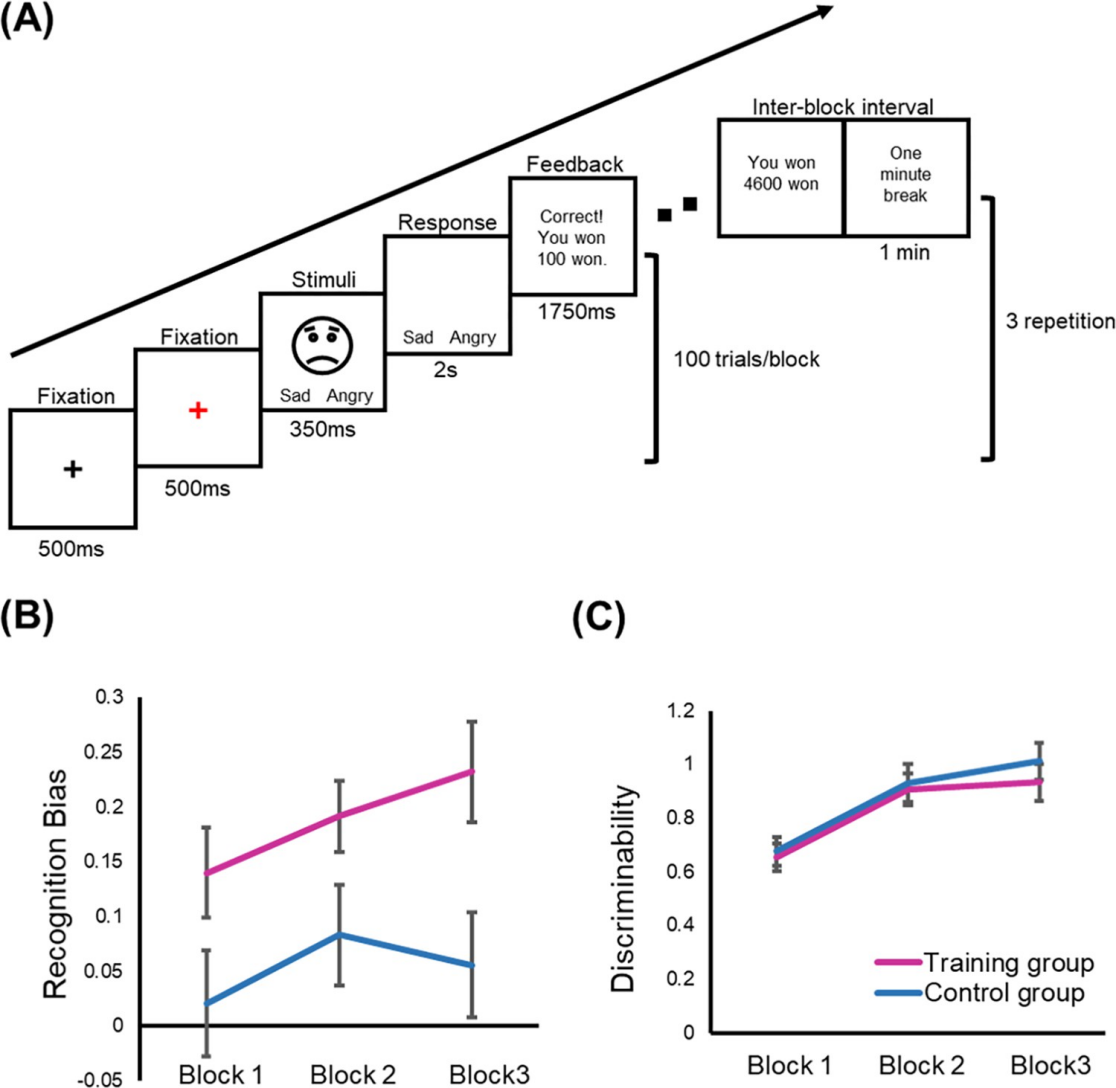
An example of a trial in the emotional PRT (A). Recognition bias (B) and discriminability (C) by group and block. The error bars represent the standard error of the means.

The intervention task consisted of three blocks of 100 trials, with a break of at least one minute between blocks. Each trial started with a black fixation cross for 500 ms. A red fixation cross appeared for 500 ms, followed by a facial expression of either 15% sadness or 15% anger for 350 ms with a presentation of word labels of “sad” and “angry” at the bottom of the screen. The word labels stayed in the screen for an additional two seconds after the disappearance of the face (Fig 1A). Participants determined the type of emotion expressed by the face by pressing ‘q’ or ‘p’ button on the keyboard. When the participants responded accurately, sometimes a reward feedback was displayed for 1750 ms, informing them that they won a monetary reward. Participants were told that not all correct trials would receive reward feedback. When participants responded incorrectly, no feedback was provided. Facial expressions were presented in a fixed pseudorandom order, and the same expression was presented not more than three times in a row. Participants were specifically instructed to try to win as much money as possible because they would receive “a portion” of the total earnings as an incentive.

The training group and control group performed the same task with different probabilistic reward assignments. For the training group, correct sad recognition received 70% reward feedback, and correct anger recognition received 30% reward feedback. This manipulation of reward probability was designed to induce recognition preference toward the sad expression. Participants were not informed of the disproportional nature of reward feedback. On the other hand, the control group received equal probabilities of a 50% reward for both correct sad and angry responses. Participants went through 10 practice trials before performing the actual task. The practice trials were equal to the actual task, but a different face model was used.

### Dot-probe attention bias task

Changes in attention bias after the intervention were assessed using the dot-probe task. We selected faces with angry (valence 2.42±0.42; arousal 4.75±0.63), fearful (valence 3.15±0.41; arousal 4.77±0.58), sad (valence 2.98±0.40; arousal 3.85±0.51), happy (valence 5.83±0.44; arousal 4.67±0.52), and neutral (valence 3.81± 0.32; arousal 2.66± 0.28) expressions from the Korea University facial expression collection (KUFEC) [29]. Normative ratings of valence and arousal were based on a 7-point scale (1 = *extremely unpleasant/not at all arousing*. 7 = *extremely pleasant/extremely arousing*).

The dot-probe attention task was created with a total of 46 pairs of faces (23 males, 23 females), consisting of each of 10 sad-neutral, fearful-neutral, angry-neutral, and happy-neutral pairs, and six neutral-neutral pairs. Faces in each pair were from the same face model, and different pairs were composed of different face models. During the dot-probe task, each trial started with a fixation cross appearing in the center of the screen for 500 ms. This was followed by the appearance of a pair of faces side by side for 500 ms. When the faces disappeared, a small dot appeared on the left or right side of the screen. Participants pressed one of two keys (“z” or “/”) to indicate whether the dot was on the left or right as quickly and accurately as possible.

Each of the 46 face pairs appeared four times, resulting in 184 total trials. The task took approximately 10 min, including a one-minute break halfway through the task. The order of face pair presentation was pseudo-random so that the same type of face pair was not presented in succession. Participants had 20 practice trials before the actual task begun.

### Empathy rating task

During this task, participants viewed a collection of video clips collected from movies and TV dramas. There were two different types of clips: emotional and neutral. Emotional clips depicted victims suffering from losses (e.g., death of a family member) or under immediate threat (e.g., a murderer’s attack). These clips were carefully selected to evoke empathy and used in a previous study [30]. Neutral clips depicted individuals experiencing ordinary life events (e.g., grocery shopping). These neutral clips were presented between the emotional clips to mitigate any potential carryover effects from the preceding emotional stimuli. Additionally, they served as individual baselines for rating responses. A total of 11 video clips were prepared (i.e., six emotional and five neutral), and each clip lasted approximately 40 sec.

Each task trial started with the presentation of a fixation cross for one second followed by short written background about the video clip (i.e., the victim as a target character, relationship between characters, antecedent context; “*A pregnant woman* *was sent to prison because her husband was killed when she tried to stop his violence*. *Not long after giving birth to a baby*, *she had to put her child up for adoption”*) for 10 sec. Then, a picture depicting a scene from the clip was shown for two seconds, during which an arrow pointed to the victim. The clip played, and participants viewed the scenes taking the perspective of the victim and imagining the scenes as real. Following the presentation of each clip, participants provided self-reported ratings on three scales. They first rated how distressed they were about the event (i.e., empathic distress) and then rated how concerned they were for the target character (i.e., empathic concern). Finally, they rated how much they wanted to help the target character featured as victim. Participants assigned the ratings on a 9-point Likert scale (1 = least *distressed/concerned/helping intentions*, 9 = most *distressed/concerned/helping intentions*) and pressed the corresponding number on the keyboard. We did not obtain help intention ratings for neutral events as the target character appearing in the neutral events did not apparently need help. After participants provided all ratings in a self-paced manner, there was a 10-sec break before moving to the next video clip.

### Self-report questionnaires

Several self-report questionnaires were obtained to ensure that participants socioemotional and empathic traits were comparable across the groups. These questionnaires included the Beck Depression Inventory-II (BDI-II) [31], the State-Trait Anxiety Inventory (STAI) [32], the state and trait categories of State-Trait Anger Expression Inventory (STAXI) [33], the Interpersonal Reactivity Index (IRI) [34], and the Prosocialness Scale for Adults (PSA) [35].

The BDI-II consists of 21 items assessing the severity of depression symptoms. Each item is rated on a 4-point Likert scale ranging from 0 to 3, with higher scores indicating greater depressive symptomatology [31]. Sample items include "I do not feel sad" to "I am so sad or unhappy that I can’t stand it." The BDI-II has demonstrated high internal consistency (Cronbach’s alpha = 0.91) and test-retest reliability (r = 0.93) [31]. The STAI consists of 40 items divided into two subscales: 20 items for state anxiety and 20 items for trait anxiety. Each item is rated on a 4-point Likert scale from 1 to 4, with higher scores indicating higher anxiety levels [32]. Sample items include "I feel calm" to "I feel tense" for state anxiety, and "I am a steady person" to "I lack self-confidence" for trait anxiety. The STAI exhibits high internal consistency (state anxiety α = 0.93, trait anxiety α = 0.90) [32]. The STAXI is a 44-item instrument assessing various dimensions of anger, including state anger, trait anger, and anger expression and control. Each item is rated on a 4-point Likert scale from 1 to 4, with higher scores indicating greater anger intensity or frequency [33]. Sample items include "I feel angry" to "I am furious" for state anger, and "I am quick-tempered" to "I am hot-headed" for trait anger. The STAXI has shown strong internal consistency (state anger α = 0.93, trait anger α = 0.86) [33].

The IRI is a multidimensional measure of empathy that includes four subscales: Perspective Taking, Fantasy, Empathic Concern, and Personal Distress. Each subscale consists of 7 items rated on a 5-point Likert scale from 0 (does not describe me well) to 4 (describes me very well). The IRI has shown good reliability, with internal consistency coefficients (Cronbach’s alpha) ranging from 0.70 to 0.78 across the subscales [34]. The PSA assesses prosocial behaviors in adults through 16 items rated on a 5-point Likert scale ranging from 1 (never) to 5 (almost always). The scale measures the frequency of prosocial actions such as helping, sharing, and comforting others. The PSA has demonstrated good internal consistency, with Cronbach’s alpha typically around 0.84 [35].

In addition, participants’ current emotional moods were also assessed using the Positive Affect and Negative Affect Scale (PANAS) [36]. The PANAS is a 20-item measure designed to assess positive and negative affect. It includes two 10-item scales: one for positive affect (PA) and one for negative affect (NA). Participants rate each item on a 5-point Likert scale ranging from 1 (very slightly or not at all) to 5 (extremely), based on how they felt during a specified time frame. The PANAS has shown good internal consistency, with Cronbach’s alphas typically ranging from 0.86 to 0.90 for the PA scale and 0.84 to 0.87 for the NA scale [36].

### Procedures

Upon arrival, participants completed a written consent form. To minimize the possibility of guessing the purpose of the study, participants were told that they would participate in two separate study sessions. Participants first instructed to complete the BDI-II [31], the STAI [32], the STAXI [33] and the the PANAS [36]. Then, they performed the emotional PRT, which lasted approximately 20 min. Then, after a short break, participants were informed that the second session had begun and were asked to complete the PANAS and STAXI-S. These two measures were used to examine any changes in mood and state anger following the intervention. Furthermore, they served as nominal cues for a separate session. Next, the dot-probe task was completed, followed by the empathy rating task. Then, the IRI [34] and the PSA [35] were obtained. Finally, participants were asked whether they thought there was a relationship between the first and second sessions of the study. When the answer was negative, they were encouraged to guess their best. Then, the participants were debriefed and thanked for their participation.

### Data reduction and statistical analyses

#### Emotional PRT

We excluded trials with reaction times less than 200 ms (1.21% of the total trials) as outliers [23]. The systematic preference for the recognition of emotion paired with the more frequent reward (i.e., sad face) was estimated following the previous methods [19, 37]. A value of 0.5 was added to each cell to allow for calculations in cases of cells with a value of 0 [37]. According to the formula, recognition bias increases as a participant correctly identifies the sad face and/or misclassifies the angry face as sad face.

Recognitionbias=0.5*log[([Sad_correct+0.5]*[Angry_incorrect+0.5])

/([Sad_incorrect+0.5]*[Angry_correct+0.5])]

We also calculated discriminability that quantifies the ability to distinguish between the sad and angry faces. High discriminability is produced by greater accuracy for both sad and angry recognition.

Discriminability=0.5*log[([Sad_correct+0.5]*[Angry_correct+0.5])

/([Sad_incorrect+0.5]*[Angry_incorrect+0.5])]

Reaction times were also assessed across the three blocks. Group differences in recognition bias and discriminability were tested with a two-sample *t*-test. Response times were analyzed separately by 2 (group) x 2 (emotion) x 3 (blocks) mixed ANOVAs. All statistical analyses in this study were conducted using IBM SPSS Statistics ver. 25.

#### Dot-probe attention bias task

Overall, we excluded 2.43% of the total trials of the dot-probe task due to incorrect responses (0.73%) and outliers (1.70%). The outliers were defined as trials with reaction times less than 200 ms or greater than three standard deviations above the individual mean [27, 30]. We assessed two subcomponents of attentional bias: orienting and disengagement. Orienting was calculated by subtracting the mean reaction times to probes replacing emotional faces in emotional–neutral pairs from the mean reaction times to probes replacing neutral faces in neutral–neutral pairs. Greater positive scores indicate faster orienting to emotional faces. Disengagement was calculated by subtracting the mean reaction times to probes replacing neutral faces in neutral–neutral pairs from mean reaction times to probes appearing in the location of the neutral expression in emotional–neutral pairs. Greater positive scores indicate slower disengagement from emotional expressions. We conducted two-way mixed ANOVAs on each attention bias index with emotion (sad vs. fearful vs. angry vs. happy) as a within-subjects factor and group (training vs. control) as a between-subjects factor.

#### Empathy rating task

Ratings obtained from each event trial were averaged for the neutral and emotional events. For the ratings of empathic concern and empathic distress, ratings specific to emotional events were assessed by subtracting scores for neutral events from scores for emotional events. Independent samples *t*-tests were conducted to reveal group differences in these adjusted ratings of empathic distress and empathic concern. Helping intentions scores obtained in the emotional event condition were also evaluated using an independent samples *t*-test.

## Results

### Demographic and self-reported questionnaires

Table 1 shows the self-reported psychological characteristics of each group and between-group test statistics. We found no group differences in age, state and trait anxiety (STAI-S and STAI-T), state and trait anger (STAXI-S and STAXI-T), depression (BDI), trait empathy (IRI), and prosocialness (PSA), *t*s < 1.1, *p*s > 0.30. Scores of positive and negative affect obtained pre- and post-training, and state anger obtained post-training did not differ either between groups, *t*s < 1.3, *p*s > 0.2.

**Table 1:** Self-reported psychological characteristics of each group and between-group test statistics.

		Training group		Control group		Test Statistics	
		(n = 35, 16 males)		(n = 36, 16 males)			
		*M*	*SD*	*M*	*SD*	*t*	*p*-value
Age		24.86	2.78	24.20	2.59	1.04	.303
STAI	State	41.67	8.89	40.51	8.15	0.57	.571
	Trait	42.92	9.03	40.91	10.66	0.86	.396
STAXI	State	12.00	4.42	12.71	5.39	−0.61	.543
	Trait	19.50	4.78	18.69	5.62	0.66	.513
BDI		8.28	5.82	7.74	7.79	0.33	.744
IRI	Personal distress	18.78	4.02	19.31	4.06	−0.56	.578
	Empathic concern	21.44	5.16	21.69	4.03	−0.22	.827
	Perspective-taking	22.92	4.58	23.43	4.67	−0.47	.642
	Fantasy	20.89	5.18	21.40	3.47	−0.49	.626
PSA		51.67	10.49	50.60	7.44	0.49	.624
PANAS-pretraining	Positive Affect	18.22	4.21	18.94	5.46	−0.62	.535
	Negative Affect	18.97	4.72	19.37	5.72	−0.32	.749
PANAS-posttraining	Positive Affect	16.39	3.51	17.71	5.62	−1.20	.236
	Negative Affect	16.72	4.94	17.86	6.12	−0.86	.392
STAXI-posttraining	State	11.78	3.28	12.46	4.25	−0.76	.453

*Note*. STAI, State-Trait Anxiety Inventory; STAXI, State-Trait Anger Expression Inventory; BDI, Beck Depression Inventory; IRI, Interpersonal Reactivity Index; PSA, Prosociality Scale for Adults; PANAS, Positive Affect and Negative Affect Scale

### Performance on the emotional PRT: Recognition bias and discriminability

Two-sample *t* tests revealed that the training group (*M* = 0.17, *SD* = 0.17) developed a greater recognition bias for sad faces compared to the control group (*M* = 0.05, *SD* = 0.22), *t* = 2.74, *d* = 0.65, *p* = .008 (Fig 1B). In contrast, perceptual discriminability did not differ between the training (*M* = 0.80, *SD* = 0.30) and control groups (*M* = 0.84, *SD* = 0.34), *t* = 0.56, *ns* (Fig 1C).

We also conducted 2 (group) x 3 (block) x 2 (emotion: sad vs. angry) ANOVA for response time. A significant main effect of block was observed, *F*(2,138) = 24.54, *η*_*p*_^2^ = 0.26, *p* < .0001. Post-hoc contrasts revealed that response times (sec) were faster in block 3 (*M* = 0.74, *SD* = 0.18) than in block 1 (*M* = 0.83, *SD* = 0.23), *F* (1,69) = 22.26, *η*_*p*_^2^ = 0.24, *p* < .0001, and in block 2 (*M* = 0.73, *SD* = 0.18) than in block 1, *F*(1,69) = 40.81, *η*_*p*_^2^ = 0.37, *p* < .0001. No difference was found between block 2 and block 3, *F*(1,69) = 0.179, *ns*. A significant main effect of emotion was observed, *F*(1,69) = 11.42, *η*_*p*_^2^ = 0.14, *p* = .001. The response times were faster for sad face recognition (*M* = 0.74, *SD* = 0.17) than for angry face recognition (*M* = 0.77, *SD* = 0.18). No main effect of group was observed, *F*(1,69) = 1.41, *ns*.

**Fig 2:**
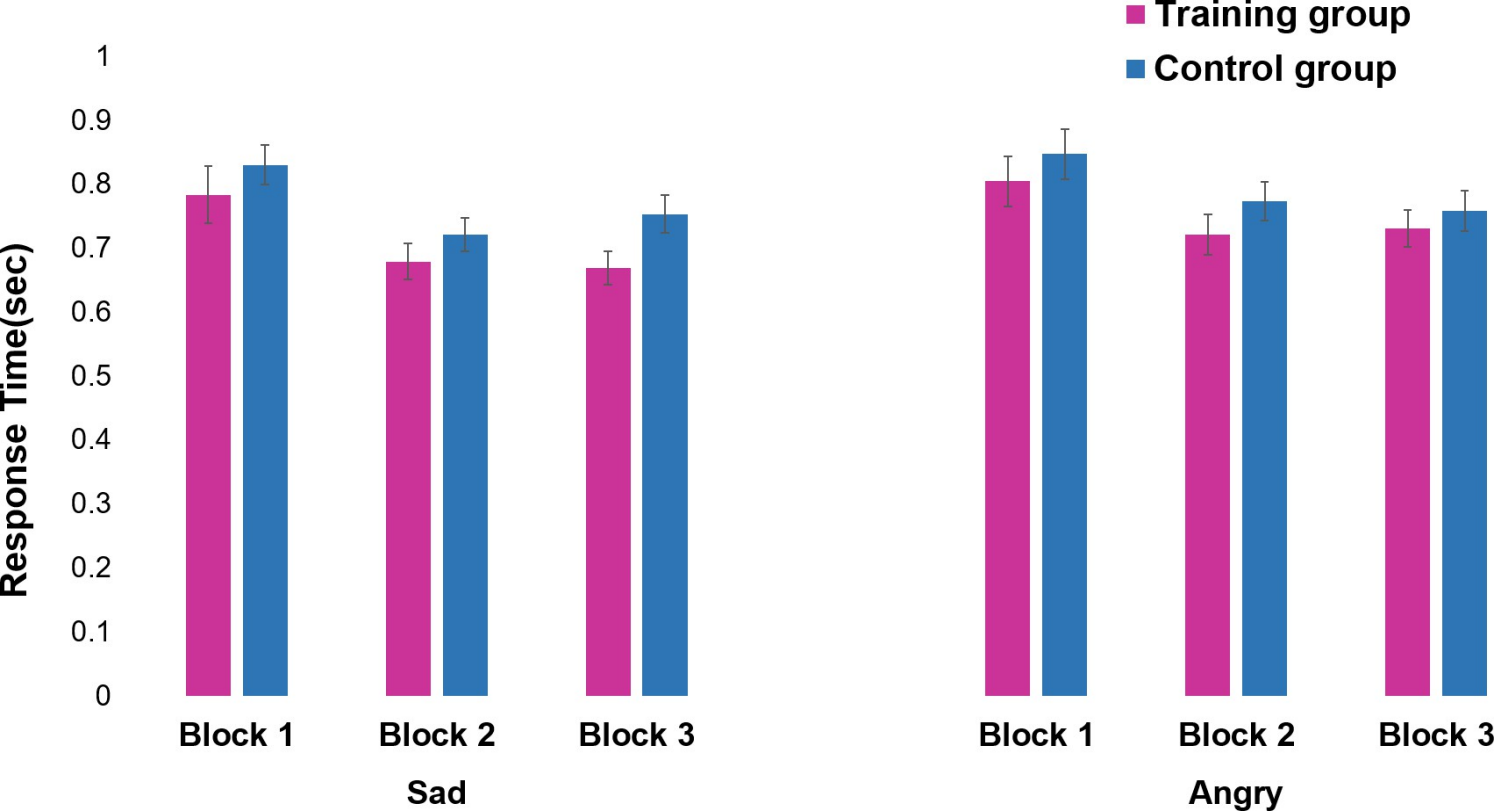
Means and standard errors of the mean response times by group and block. The error bars represent the standard errors of the means.

There was a significant three-way interaction, *F*(2,138) = 4.68, *η*_*p*_^2^ = 0.06, *p* = .011. To disentangle the interaction effect, 2 (group) x 2 (emotion) ANOVAs were conducted separately for each block. For block 1, there was no main effect of emotion, *F*(1,69) = 1.90, or group, *F*(1,69) = 0.69, and no interaction effect, *F*(1,69) = 0.02. For block 2, there was a main effect of emotion, *F*(1,69) = 16.96, *η*_*p*_^2^ = 0.20, *p* < .0001, with faster response times for sad faces (*M* = 0.70, *SD* = 0.16) than for angry faces (*M* = 0.75, *SD* = 0.19). We found no main effect of group, *F*(1,69) = 1.39, or interaction effect, *F*(1,69) = 0.22. For block 3, we found a significant interaction effect of group and emotion, *F*(1,69) = 7.64, *η*_*p*_^2^ = 0.10, *p* = .007 (Fig 2). Post-hoc *t* tests revealed that sad recognition was faster in the training group (*M* = 0.67, *SD* = 0.16) than in the control group (*M* = 0.75, *SD* = 0.18), *t* = 2.15, *d* = 0.51, *p* = .035. The response time for anger recognition did not differ between the training (*M* = 0.73, *SD* = 0.17) and control groups (*M* = 0.76, *SD* = 0.19), *t* = 0.64, *ns*.

### Measurement tasks

#### Dot-probe task

Table 2 shows the means and standard deviations of the attentional orienting and disengagement scores for each emotion across groups. A 2 (group) x 4 (emotion) ANOVA on attentional orienting revealed a significant main effect of emotion, *F*(3,207) = 6.02, *η*_*p*_^2^ = 0.08, *p* = .001. Post-hoc contrasts revealed that overall orienting scores were greater for happy faces than for fearful faces, *F*(1,69) = 10.42, *η*_*p*_^2^ = 0.13, *p* = .002, and sad faces, *F*(1,69) = 14.19, *η*_*p*_^2^ = 0.17, *p* < .0001. The orienting scores for angry faces were greater than those for sad faces, *F*(1,69) = 7.04, *η*_*p*_^2^ = 0.09, *p* = .010. There were no differences between sad and fearful faces, *F*(1,69) = 1.10, angry and fearful faces, *F*(1,69) = 2.92, or angry and happy faces, *F*(1,69) = 1.12. No main effect of group or interaction effect was revealed, *Fs* < 0.09.

**Table 2:** Means and standard deviations of attentional orienting and disengagement scores by group and emotional face type.

		Training group		Control group	
		*M*	*SD*	*M*	*SD*
Orienting	Angry face	3.60	13.16	4.86	17.44
	Fearful face	0.70	10.90	0.50	16.43
	Happy face	5.80	14.90	6.99	15.26
	Sad face	−1.89	12.71	−1.24	18.62
Disengagement	Angry face	−0.54	14.72	0.29	17.54
	Fearful face	−1.00	13.39	1.26	18.89
	Happy face	−9.63	10.69	−6.63	17.71
	Sad face	−7.03	12.86	−3.40	16.15

A 2 (group) x 4 (emotion) ANOVA on attentional disengagement revealed a significant main effect of emotion, *F*(3,207) = 8.23, *η*_*p*_^2^ = 0.11, *p* < .0001. Post-hoc contrasts revealed that disengagement scores were greater for angry faces than for happy faces, *F*(1,69) = 14.54, *η*_*p*_^2^ = 0.17, *p* < .0001, and sad faces, *F*(1,69) = 5.19, *η*_*p*_^2^ = 0.07, *p* = .026. The disengagement scores for fearful faces were greater than for happy, *F*(1,69) = 22.74, *η*_*p*_^2^ = 0.25, *p* < .0001, and sad faces, *F*(1,69) = 7.50, *η*_*p*_^2^ = 0.10, *p* = .008. No differences were found between sad and happy faces, *F*(1,69) = 2.62, or between angry and fearful faces, *F*(1,69) = 0.02. There was no main effect of group or group x emotion interaction, *Fs* < 0.8.

**Fig 3:**
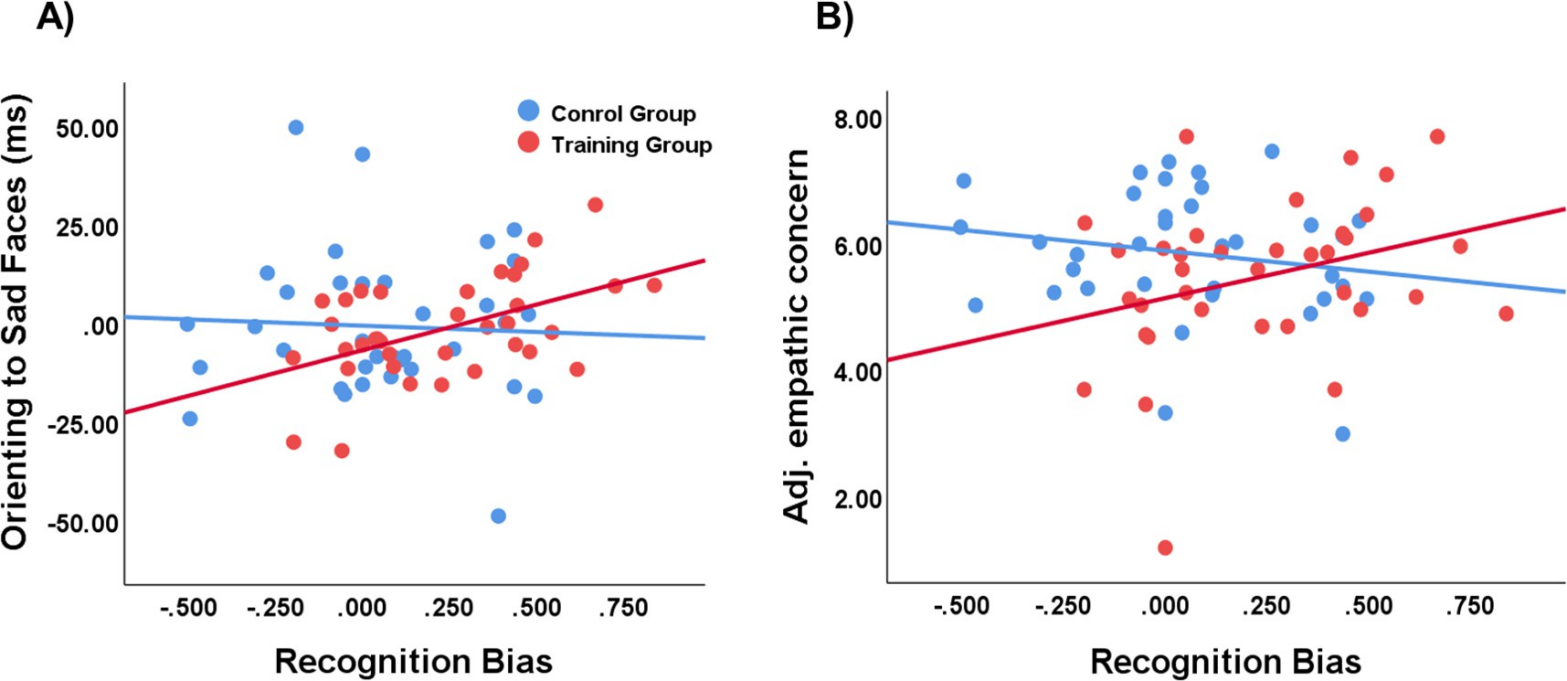
Scatter plots showing a significant positive association between recognition bias and orienting to sad faces (A) and empathic concern (B) in the training group but not in the control group.

We further examined whether the learned recognition bias for sad faces through the emotional PRT predicted attentional bias to sad and angry faces. We conducted moderation analyses using the PROCESS macro v3.5 [38]. The PROCESS procedure provides bootstrap confidence intervals to test for significance. An effect is considered significant if the 95% confidence interval (CI) does not contain zero [39]. Recognition bias was entered as an independent variable, with orienting and disengagement scores as dependent variables, and group as the moderator (training group coded as 1, control group coded as -1). Notably, the learned recognition bias was calculated using data from the final block (Block 3) rather than from all blocks. This approach was chosen because the data from Block 3 more accurately reflects the recognition bias acquired through the intervention. With orienting scores for sad faces as the dependent variable, results indicated the presence of a moderation effect of training, *b* = 13.16, *SE* = 6.66, *t* = 1.97, *p* = .052. Simple slope analyses revealed that recognition bias predicted faster orienting to sad faces in the training group, *b* = 23.13, *SE* = 9.47, 95% CI [4.23, 42.04], *t* = 2.44, *p* = .017, but not in the control group, *b* = −3.18, *SE* = 9.38, 95% CI [−21.91, 15.54], *t* = −0.34, *ns* (Fig 3A). With the disengagement scores for sad faces as the dependent variable, we did not find significant moderation effect, *b* = −9.85, *SE* = 6.26, *t* = −1.57, *ns*. Similarly, moderation analyses with the orienting and disengagement scores for angry faces revealed no statistically significant moderation effects, *b* = 3.22, *SE* = 6.67, *t* = .48, *p* = .63 for orienting, and *b* = −9.50, *SE* = 6.91, *t* = −1.37, *p* = .17 for disengagement, respectively.

For a better understanding of moderation analysis results, we conducted a sensitivity analysis to estimate the minimum detectable effect size, given our sample size. The current sample size was determined to detect effects in group differences on task outcomes. With the current sample size of 71, an alpha level of 0.05, a power of 0.80, and three predictors (independent variable, moderator, and their interaction term), G*Power software version 3.1.9.4 [24] calculated a minimum effect size of Cohen’s *f*^2^ = .11 to be reliably detected in our sample. This effect size falls between the medium and large range [40].

#### Empathy rating task

Table 3 presents the means and standard deviations of empathic distress, empathic concern, and helping intentions across groups. Independent samples *t*-tests revealed no group differences in the adjusted empathic distress (training group, *M* = 4.63, *SD* = 1.42; control group, *M* = 4.84, *SD* = 1.21), *t* = 0.66, *ns*, adjusted empathic concern (training, *M* = 5.45, *SD* = 1.25; control, *M* = 5.82, *SD* = 1.02), *t* = 1.39, *ns*, and helping intentions scores (training, *M* = 6.62, *SD* = 1.42; control, *M* = 7.17, *SD* = 1.09), *t* = 1.85, *ns*.

**Table 3:** Means and standard deviations of empathic distress, empathic concern, and helping intentions by group and condition.

Empathy rating task		Training group		Control group	
	Condition	*M*	*SD*	*M*	*SD*
Distress	Emotional clip	6.44	1.48	6.42	1.25
	Neutral clip	1.82	0.72	1.59	0.63
Concern	Emotional clip	7.30	1.05	7.46	0.95
	Neutral clip	1.85	0.70	1.64	0.58
Helping intentions	Emotional clip	6.62	1.42	7.17	1.09

We further examined whether the acquired recognition bias for sad faces predicted empathic rating scores. Similar to moderation analyses with attention bias scores, recognition bias at the last block entered as the independent variable, with adjusted empathic concern and empathic distress ratings entered as the dependent variables, and group as the moderator. We found a significant interaction effect with empathic concern scores, *b* = 1.04, *SE* = .48, *t* = 2.17, *p* = .03, indicating the presence of a moderation effect of training (Fig 3B). Simple slope analyses revealed that recognition bias predicted increased empathic concern ratings in the training group, *b* = 1.43, *SE* = .69, 95% CI [.06, 2.80], *t* = 2.09, *p* = .04, but not in the control group, *b* = −.66, *SE* = .68, 95% CI [−2.01, .69], *t* = −0.97, *ns*. Whereas, there was no moderation effect with empathic distress ratings, *b* = .62, *SE* = .56, *t* = 1.10, *ns*.

## Discussion

In the current study, we developed an emotional version of the PRT that facilitated recognition of sadness over anger from ambiguous facial expressions. We found that individuals who developed greater preference for sad recognition during the emotional PRT showed faster attentional orienting toward sad faces and increased empathic concern toward distress others. These results suggest the potential of the emotional PRT for modulating socioemotional processing.

This study demonstrates that probabilistic reward learning can be applied to facial emotion recognition, successfully inducing recognition bias for an emotional expression associated with a more frequent reward. As correct sadness recognition received more frequent rewards during the emotional PRT, participants in the training group identified sadness more frequently from ambiguous facial expressions of sadness and anger. Unlike recognition bias, however, there was no group difference in discriminability. This observation indicates that the two groups developed comparable sensitivity to detect visual perceptual differences between the two facial expressions [3], which is consistent with the view that perceptual sensitivity can be developed as a function of simple exposure regardless of task relevance [41]. In contrast, biased recognition decisions can be obtained by task-dependent reinforcement. According to the incentive salience hypothesis [42], providing a reward for a response makes the stimulus associated with the response perceptually and motivationally more salient, leading to a stronger association between the stimulus and response. Thus, sadness facial cues from ambiguous expressions may have become more salient during the emotional PRT. This increased saliency of sadness cues accounts not only more frequent recognition of sadness but also faster responses for sad recognition observed at the last learning block in the training group than in the control group.

Contrary to our expectations, there were no statistical group-level differences in attentional orienting and disengagement following the emotional PRT. Nonetheless, participants who developed greater sad recognition bias following the effective training showed increased attentional orienting to sad faces. The significant association between attentional bias and recognition bias within the training group suggests that acquired recognition bias during training can contribute to early attentional processing of facial expressions of emotions. This finding is consistent with previous research showing a close association between recognition and attentional resource allocation processes of emotions [21].

Similarly, despite the absence of group effect, the acquired sad recognition bias in the training group accounted for greater empathic concern towards distressed others, as indicated by the moderation analysis results. These results are partly consistent with previous research demonstrating that explicit attentional engagement with sad faces can enhance empathic and prosocial responses [14, 15]. It is noteworthy that the positive association between acquired sad recognition bias and empathic concern in the training group did not extend to empathic distress. Both empathic concern and empathic distress are recognized as the two key components of empathy; nevertheless, they may involve distinct mechanisms and play different functional roles in relation to prosocial responses [17, 43]. Together, these findings of distinct associations suggest that acquired recognition bias for a specific emotion through emotional PRT may exert a targeted influence on early attentional processing for facial emotions and empathic responses toward distressed others. Regarding the lack of group differences in the current study, we speculate that one session of the emotional PRT may not have been sufficient to induce recognition bias strong enough to generate group-level differences in outcome measures. Previous research has suggested that implicit task-based interventions may be more efficient with more training sessions [44, 45]. Future studies should explore the application of multiple sessions of emotional PRT.

In the current study, we found that a single session of the emotional PRT successfully induced a recognition bias towards sad facial expressions, subsequently modulating related socioemotional processing. While this study presents promising results in the first attempt to use an emotional version of the probabilistic reward task to induce recognition bias for an emotion, there are limitations. First, we assessed postintervention outcomes only. Although the random assignment of participants to either group ensured no group differences in the mood- and empathy-related trait measures, there might be individual differences in learning through the emotional PRT. Future studies assessing pre- to post-intervention differences will help to clarify the specific contribution of the emotional PRT across participants to the effects found in this study. Second, we failed to identify statistically significant group-level differences in attention and empathy related outcomes. Although one session of the emotional PRT successfully induced recognition bias within the training context (i.e., near transfer), it may require repeated and consistent training to generate robust group-level effects in cognitive domains outside of training context (i.e., far transfer) [46]. Furthermore, notably, the control group was also rewarded for the correct sad recognition but only with reduced probability compared with the training group. Thus, group-level differences due to the emotional PRT would have been less pronounced than when no reward was provided for correct sad recognition in the control group. Third, the current study targeted to modify sadness recognition using the emotional PRT. Further research is needed to determine whether the emotional PRT can be effective in modifying the recognition of other facial emotions. Fourth, the current study assessed the short-term effects of the emotional PRT only. Whether and how long these effects persist is unknown and requires follow-up studies. Finally, we note that the nature of induced recognition bias through the emotional PRT needs further investigation. Although we observed clear evidence of more frequent recognition of sadness after the emotional PRT, it remains unclear whether this increased sad recognition merely reflects a strategic decision to prefer sad recognition or is accompanied by facilitated sad cue processing. We believe that the close association between induced recognition bias and attentional orienting to sad faces supports the latter; however, a systematic investigation is warranted for a clearer understanding on this matter. Future studies can examine the causal chain of psychological processes induced by the emotional PRT using experimental mediation approaches [47, 48]. In addition, an important future direction could be the use of neuroimaging methods to examine changes in the neural correlates of emotional and cognitive processes in combination with the emotional PRT.

Overall, the current study highlights the potential of the emotional PRT in successfully inducing recognition bias for facial expressions of emotions. Despite the limitations of the study, the results show that the emotional PRT is effective in inducing recognition bias for facial emotions. The emotional PRT is a simple and easy-to-implement intervention. It can be adapted to meet varying individual needs and has the potential to be a useful intervention for clinical and subclinical groups characterized by maladaptive biased recognition of emotions. Further research is needed to investigate the emotional PRT in inducing recognition bias for other facial emotions and to test its efficacy in different populations.
